# Two *Doublesex1* mutants revealed a tunable gene network underlying intersexuality in *Daphnia magna*

**DOI:** 10.1371/journal.pone.0238256

**Published:** 2020-08-31

**Authors:** Quang Dang Nong, Tomoaki Matsuura, Yasuhiko Kato, Hajime Watanabe

**Affiliations:** 1 Department of Biotechnology, Graduate School of Engineering, Osaka University, Suita, Osaka, Japan; 2 Biotechnology Global Human Resource Development Program, Division of Advanced Science and Biotechnology, Department of Biotechnology, Graduate School of Engineering, Osaka University, Suita, Osaka, Japan; 3 Frontier Research Base of Global Young Researchers, Graduate School of Engineering, Osaka University, Suita, Japan; Shanghai Ocean University, CHINA

## Abstract

In recent years, the binary definition of sex is being challenged by repetitive reports about individuals with ambiguous sexual identity from various animal groups. This has created an urge to decode the molecular mechanism underlying sexual development. However, sexual ambiguities are extremely uncommon in nature, limiting their experimental value. Here, we report the establishment of a genetically modified clone of *Daphnia magna* from which intersex daphniids can be readily generated. By mutating the conserved central sex determining factor *Doublesex1*, body-wide feminization of male daphniid could be achieved. Comparative transcriptomic analysis also revealed a genetic network correlated with *Doublesex1* activity which may account for the establishment of sexual identity in *D*. *magna*. We found that Dsx1 repressed genes related to growth and promoted genes related to signaling. We infer that different intersex phenotypes are the results of fluctuation in activity of these Dsx1 downstream factors. Our results demonstrated that the *D*. *magna* genome is capable of expressing sex in a continuous array, supporting the idea that sex is actually a spectrum.

## Introduction

Sexual dimorphism is common and widespread trait of animals. Although the processes of sexual development, i.e. how sex is determined and how sex-specific traits are established, are greatly diverse among species, sex has been recognized to be classified as two categories, male and female [1]. However, the rule of dimorphism does not always prevail. To date, there have been numerous records of individuals that cannot be classified as male or female across various animal groups ranging from crustaceans [2], insects [3], birds [4], fish [5], to mammalians [6] including human [7]. Such individuals are either sexually mosaic, meaning male and female characteristics of any categories (morphology, gonad or even behavior) coexist in the same body, or in other cases are intermediate uniformities between male and female. This seemingly natural existence of sexual ambiguities in any populations implies that the binary view of sex needs to be revised [8].

Sexual phenotypes deviating off the typical male and female scope can be the result of particular genetic and/or non-genetic conditions. In nematode, fly and human, numerous variations of untypical sexual characteristics caused by small mutations have been documented [9–11]. Temperature or bacterial infection can alter the sex of many insects [12,13]. Fish is also an animal group whose sex is plastic and subjected to changes in temperature, pH, hypoxia, population density, etc [14,15]. For animals utilizing sex hormones like most vertebrates, although the sex determination cascade is strictly controlled by genetic machinery, they are very sensitive to hormone analogs and endocrine disrupting chemicals (EDCs) that may present in their surroundings and end up developing phenotypes that are inconsistent with their genotypic sex [16]. To address sexual ambiguities without regard for the underlying cause, a neutral umbrella term, “intersex,” is often used. Intersex encompasses numerous variations of sexual characteristics, suggesting that there are much more than just male and female in nature, or in another word, the biological sex is a spectrum.

To justify the spectrum model of sex, top-down study using intersex animals is of great importance. In the past, by analyzing mutants showing abnormal sexual dimorphism in *Drosophila melanogaster* and *Caenorhabditis elegans*, sex determining genes were identified [17]. We can expect that similar approach in other species will lead to the findings of more key factors related to sexual dimorphism and eventually shed light on the molecular makeup underlying sexual identity. However, intersexuality occurs at very low frequency, making it difficult to use them in exhaustive experiments.

Accumulating studies into the molecular pathway of sex determination have revealed that, despite the great diversity at the top of sex determination cascade and heavily branching of sexual differentiation mechanism, a common node can be found at the connecting point of the two processes where the DM-domain gene family is involved [18,19]. This family of transcription factors contains a DNA-binding domain that is conserved throughout the animal kingdom, known as the DM domain. Binary activity of DM-domain genes, either alternative-splicing style or on/off style, was found to play an important role in regulating the sexual development of many species. The alternative-splicing style is found in insects where the DM-domain gene *Doublesex* (*Dsx*) can be spliced in to either male of female isoforms, each version will in turn promote the differentiation into one sex and repress the opposite [20]. The on/off style is found in many other species including mammalians, birds, reptiles, fish, and nematode… where activation of DM-domain genes generally results in male differentiation [21,22]. Although different animal groups recruit very different machinery to control the activity of this sex determining gene, the ultimate goal is to stabilize its binary behavior, suggesting that binarism of the sex-determining switches is very important in the creation of sexual dimorphism.

However, unlike genes in the sex-determination cascade, those of sexual differentiation network which directly give rise to sexual traits do not seem to follow the binary law. Many dimorphic characteristics, for example body size, muscle mass or even circulating hormonal level, are quantitative traits which is affected by both genetic and environmental factors and usually display overlap between male and female. Interestingly, it was reported that in *D*. *melanogaster*, intersex phenotypes could be obtained from *dsx* (the DM-domain gene identified in this species) mutants in which this gene was completely or partially disrupted [23]. These data suggest that although important, binary action of sex determining switches like the DM-domain gene alone is insufficient for assuming the outcome of sex. More similar data from other species would be necessary to clarify the role of the molecular cascade where DM-domain genes are involved in the establishment of sex.

The water flea *Daphnia magna* is a freshwater crustacean that is well known for their ability to undergo cyclic parthenogenesis [24]. While female *D*. *magna* produces clonal daughters asexually by default, they can also produce clonal sons in response to environmental cues such as lack of food, shortened day length, and high population density [25,26]. In response to those stimuli, females also change their reproductive mode from parthenogenesis to sexual reproduction [27]. In another word, the same genetic information can lead to three reproductive forms, either asexual female, sexual female or sexual male, all responsively to epigenetic factors, making them a very interesting model for the study of sex. Noticeably, previous studies have showed that on/off activity of a DM-domain gene called *DapmaDsx1* (or just *Dsx1*) plays an important role in male determination of *D*. *magna* [28].

Here, we report that by modifying the *Dsx1* locus in *D*. *magna* using a genome editing tool, we were able to generate and maintain two different mutated genotypes from which feminized males can be consistently generated. In one strain (which has already been published [29]), mono-allelic knock-out of *Dsx1* causes minor feminization in males but still retains their ability to fertilize eggs. In another strain, knock-out of one allele and deletion of the start codon of the opposite allele resulted in severe feminization and disrupted male reproduction ability. Transcriptomic analysis using these intersex strains also revealed a collection of genes that are correlated with the reduced Dsx1 activity, implying that in *D*. *magna*, the sexual differentiation cascade can be finely tuned to display sex in a spectrum rather than just typical male and female.

## Results

### Generating two *Dsx1* mutants by genome editing

**Fig 1:**
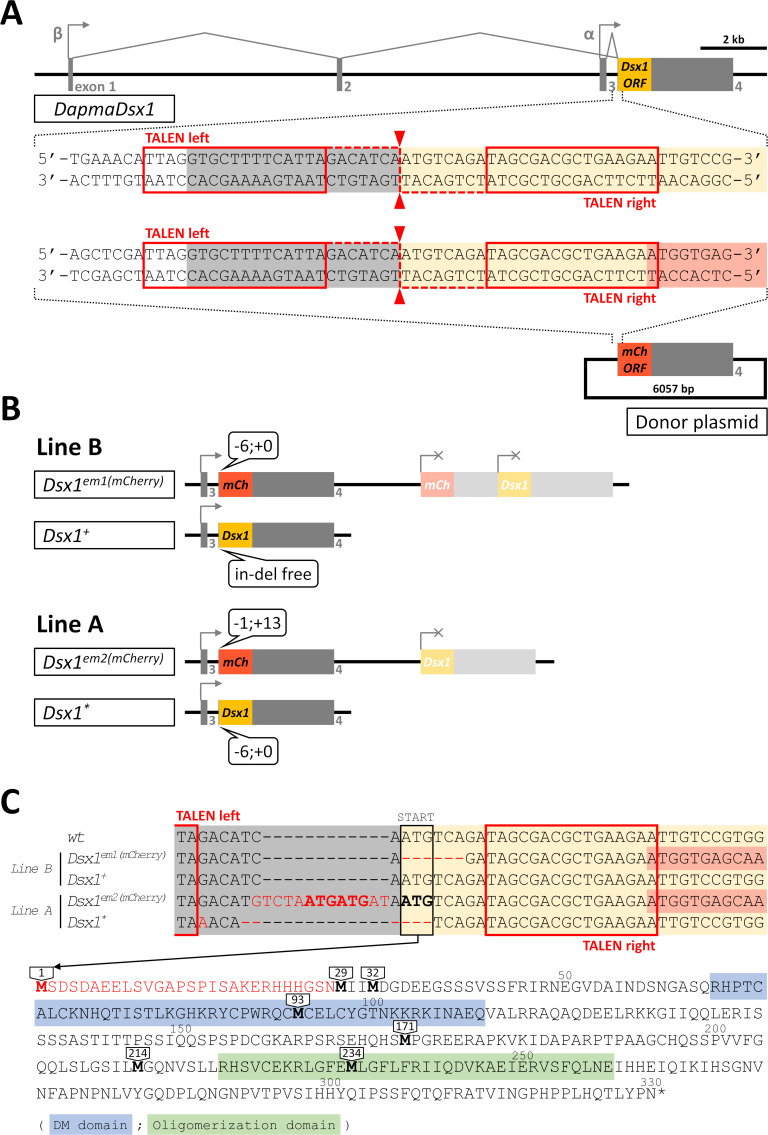
Genotype of the two *Dsx1*-related mutants. **A)** Structure of *Dsx1* gene of *Daphnia magna* and the chosen design of TALEN for genome editing. There are two different promoters, alpha and beta, directing the transcription of two *Dsx1* transcripts which are different only in 5’ UTR region. TALEN was designed to target a 46 bp sequence at the beginning of the common exon 4 which contains the full ORF of *Dsx1*. Cleavage is expected to occur right in front of *Dsx1* start codon. A donor vector carrying a copy of promoter-less *mCherry* was also used in this targeting strategy. The 46-bp TALEN target sequence is cloned into 5’ end and the full-length 3’ UTR of *Dsx1* is cloned into 3’ end of this *mCherry* to allow NHEJ-mediated knock-in so that *mCherry* can replace *Dsx1* ORF and report the activity of the locus. **B)** Diagram illustrating the genotype at *Dsx1* locus of the two mutant strains, Line A and Line B. Both are hemizygous knock-ins with in-del mutations found at TALEN cleavage site. **C)** Nucleotide sequence of in-del mutations from the four alleles shown in panel B). At the bottom, the full amino acid sequence of Dsx1 protein is shown together with the position of in-frame methionines that may become the alternative start codon in case the original one is deleted.

We had previously reported the creation and characterization of a ‘*Dsx1* reporter strain’ which was obtained by TALEN-mediated knock-in of an *mCherry* reporter via non-homologous end joining [29] (see Fig 1A for a description of targeting strategy). This ‘*Dsx1 reporter strain*,’ referred to as ‘Line B’ in this paper, has one intact *Dsx1* allele and another mutated allele harboring *mCherry* reporter.

At the same time with the creation of ‘Line B,’ by the same approach, we also had reported the generation of another transgenic line, which we named ‘Line A.’ In current study, we performed genotyping of Line A and found that on one *Dsx1* allele, a 6-bp deletion occurred at the target site of TALEN, removing the start codon ATG of *Dsx1* ORF (Fig 1B and 1C), while the another allele of Line A contained one copy of *mCherry* reporter. As parthenogenetic females of both mutants could produce their offspring quite normally, we were able to maintain the hemizygous genotypes of Line A and Line B through asexual cycle of the population.

### Difference of intersex phenotypes between the *Dsx1* mutants

We had previously reported that Line B shows minor feminization at non-gonadal tissues in which little shift in morphology of male specific structures toward female appearance is detected. Although reduction in mating ability was also found, Line B males could still successfully fertilize and create viable resting eggs [29].

**Fig 2:**
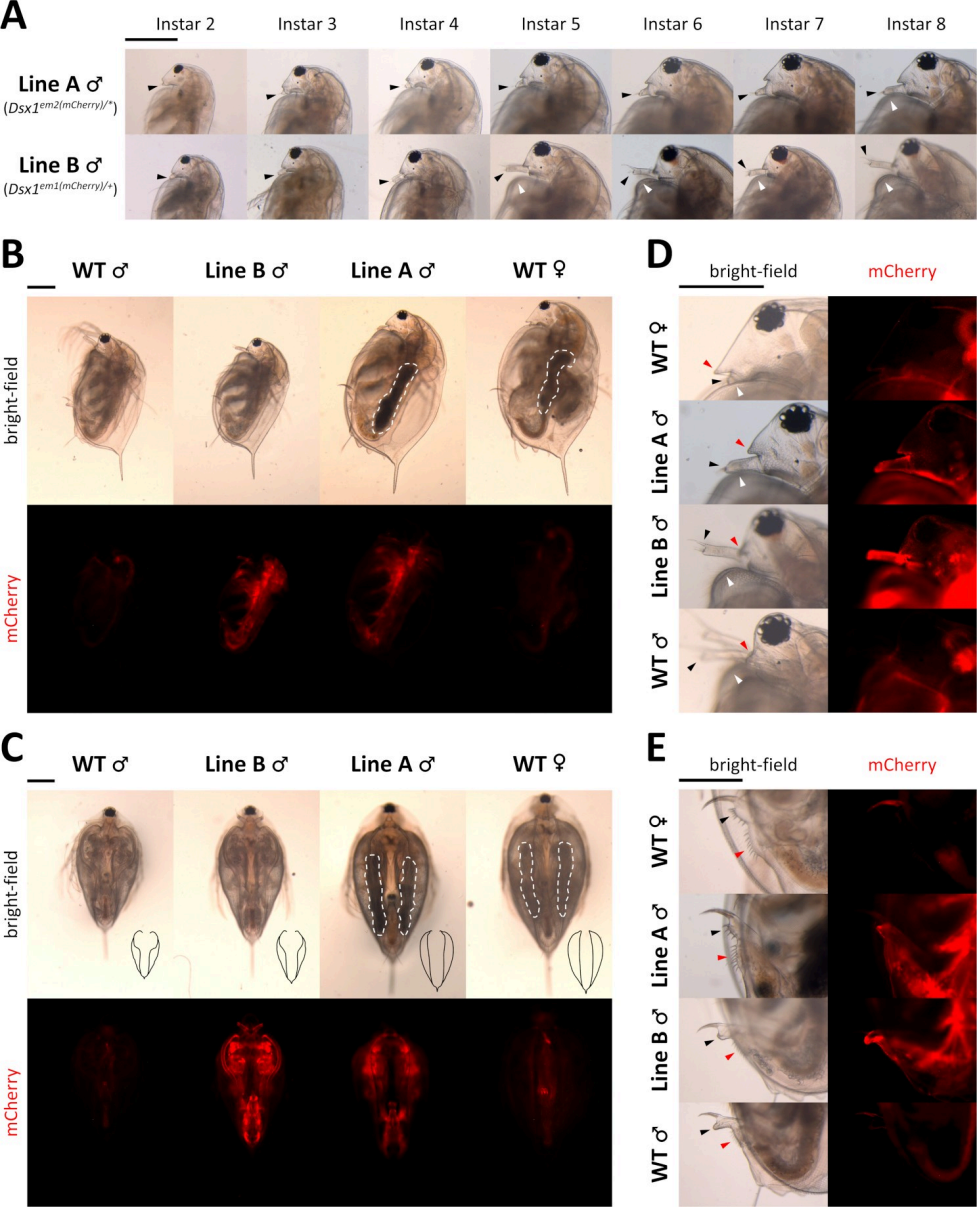
Feminization in the phenotype of *Dsx1* mutants. **A)** Head region with focus on the first pair of antennae of Line A males and Line B males of various ages. Until instar 4 is the juvenile stage of daphniids. Transition to fully mature adults usually occurs when the animal reaches instar 5, which is approximately one week after birth. Black-arrow heads: first antennae. White arrowheads: carapace curve that reveals copulation hooks in males. **B)** Full body, side view of instar 8 daphniids. White dotted lines indicate ovary-like gonads, which appeared darker because of the accumulation of yolk protein. **C)** Full body, ventral view of instar 8 daphniids. Black drawings at bottom right corner of each bright-field photos are tracing lines scaled down to 1:2.26 ratio depicting the outline of the carapace edge. White dotted lines have the same meaning as in panel B. **D)** Head region, side view of instar 8 daphniids. Black and white arrowheads have the same meaning as in panel A. Red arrowheads indicate the rostrum. **E)** Genital and anal region, side view of instar 8 daphniids. Black arrowheads: genital. Red arrowheads: anus. Each panel from A to E has one scale bar indicating 0.5 mm, which is shared among all photos of the same panel.

To examine the effect of Line A genotype on sexual differentiation, we observed phenotypes of males that were produce by fenoxycarb exposure as described previously [30]. In Line A, severe feminization took place from a very early stage of development and persisted until adulthood. In particular, the first pair of antennae of Line A male showed obvious elongation which is a distinctive trait of male daphniids. However the length was short comparing to Line B male or wildtype male. This difference was visible in juveniles (instar 2–4) and became even more obvious after puberty (instar 4 to instar 5) when Line B male quickly transformed to adult morphology but Line A male showed very little change (Fig 2A). At adulthood, feminization effect was even more profound in many morphology aspects. In details, Line A male had average body length comparable to wildtype female, which is roughly 25% larger than normal male (Fig 2B and 2C). The ventral opening of the carapace in Line A male did not have the revealing curve around the first body segment as typically found in male but was rather straight and closed like female (Fig 2C). At the rostrum region, aside from the barely elongated first antennae, helmet of Line A male also showed female-like bulky appearance but not the closely fit shape of male (Fig 2D). Furthermore, Line A male seemed to have the genital and anus similar to those of female (Fig 2E).

Especially, upon reaching the age of sexual maturity, irregular gonad development could be observed in Line A males. Typically, testes of male daphniids are transparent and thus impossible be recognized by naked eyes. On the other hand, ovaries of females are noticeably darkened due to the accumulation of yolk protein. In Line A male, ovary-like darkened gonads were found in all cases. However, no eggs were released from these ovary-like structures even after several weeks of culturing, making the gonads of Line A male remain thick and dark despite multiple times of molting (Fig 2B and 2C).

From this observation, we categorized males of Line A and Line B as intersexes, in which Line B male exhibited mild feminization while Line A male displayed more severe transformation to female. Assuming that *D*. *magna* sex is a spectrum with typical female and male occupying the two ends, Line A male and Line B male would represent two different positions in the middle of the scale.

### Expression of *Dsx1* mRNA in the mutants

**Fig 3:**
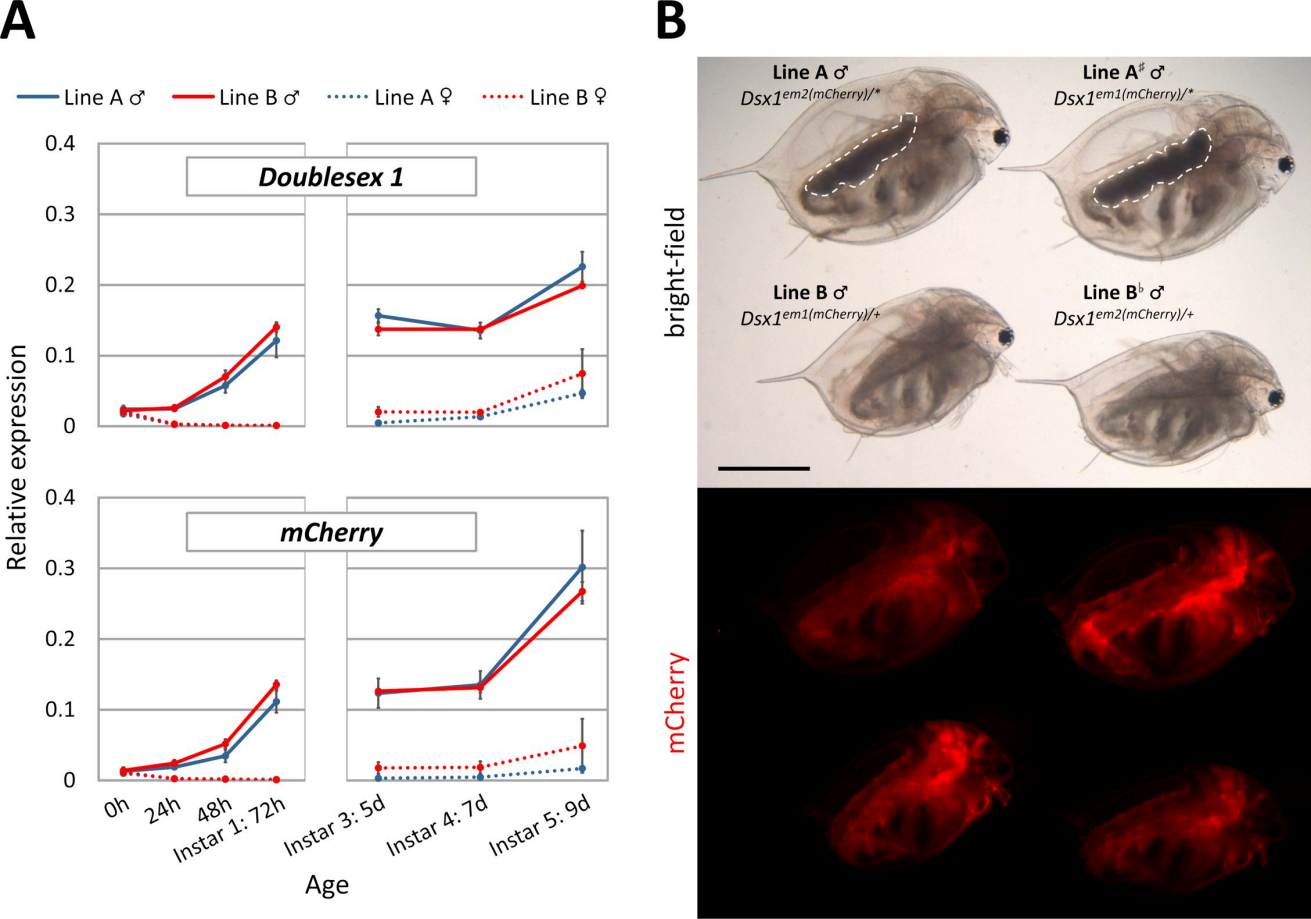
**A)** Whole body mRNA level quantified by qPCR targeting the two alleles at *Dsx1* locus. Expression of *L32* gene was used for normalization. **B)** Difference in mCherry intensity between Line A, Line B and two hybrid lines obtained from a crossing between Line A and Line B. All four daphniids are of the same age (instar 8, 17 day-old) and photographed in one single photo to allow direct comparison. White dotted lines indicate the ovary-like darkened gonads. Scale bar indicates 1.0 mm.

As previously reported, in Line B male, mRNA level of *Dsx1* was roughly half of that of wildtype male, possibly because of the lack of one *Dsx1* copy [29]. To confirm the effect of mutated *Dsx1* allele in Line A on its own expression, we performed quantitative PCR using whole body samples of different ages. We found that in Line A, the dimorphic patterns of *Dsx1* and *mCherry* expression were similar to those in Line B without any statistical difference (Fig 3A).

Despite the same mRNA level of *mCherry*, while observing the fluorescence signal, we noticed that fluorescence intensity in the males of Line A was slightly weaker comparing to those of Line B (Line A vs. Line B, Fig 3B). To confirm whether this was because of the introduction of in-frame ATGs to the upstream of start codon of *mCherry* in Line A (Fig 1C), we performed a cross using Line A females and Line B males to swap the two *mCherry* alleles. As a result, there was no difference in mCherry fluorescence between the two mutants if both had the same *mCherry* reporter sequence. We also found that the *mCherry* allele derived from Line B had better performance probably due to no upstream ATG (Line A^#^ and Line B vs. Line A and Line B^b^, Fig 3B).

Despite the difference in feminization level, it could be seen from these results that there was no change in transcription, translation, as well as spatiotemporal control of the *Dsx1* locus (Fig 2, mCherry photos) between Line A male and Line B male. These data suggested that the cascade relaying male determination signal from fenoxycarb to Dsx1 was not disrupted by the 6-bp deletion at start codon ATG of *Dsx1* in Line A, and that only genes downstream of *Dsx1* in the pathway were affected.

### Non-binary global gene expression along sex spectrum in 40 h embryos

Since Dsx1 is a transcription factor, its direct and indirect target genes must have been affected transcriptionally in the mutants. Therefore, in order to characterize this shift in transcriptome of intersex animals, RNA sequencing approach was used. 40-hour embryos of wildtype female, Line A male, Line B male and wildtype male were chosen as representatives of four different positions in *D*. *magna* sex spectrum for this experiment. We targeted the stage of 40 hours post oviposition (hpo) for two main reasons. Firstly, gene expression in older daphniids may fluctuate greatly under the influence of molting cycle and environmental factors. It is much easier to obtain synchronized samples from embryos in which the Dsx1 cascade would stand out more without noises from other gene networks. Secondly, according to the phenotyping result, morphological difference was obvious even in first swimming juvenile (72 hpo), indicating that segregation of sex differentiation cascade had already occurred during organogenesis (16–24 hpo).

**Fig 4:**
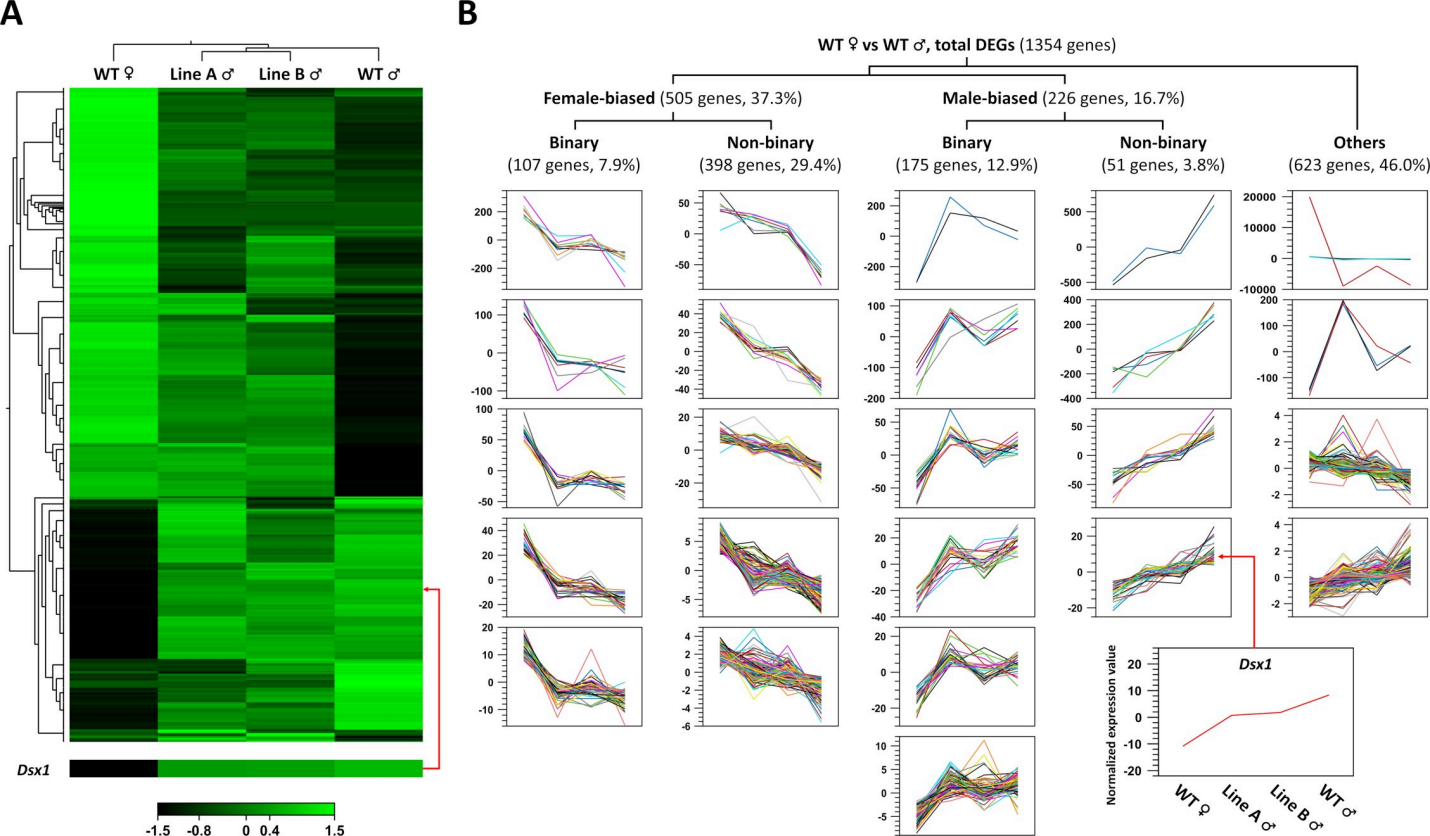
**A)** Heat map of 1354 differentially expressed genes detected in the wild type female vs. wild type male comparison (EDGE-test). Each row represents one gene. In addition to wild type female and wild type male gene expression (left-most and right-most column), intersex gene expression is also shown (two columns in the middle). A close-in for *Dsx1* together with an arrow leading back to its original position is provided at the bottom of the heat map. Color scale indicates normalized logCPM values in which brighter green means stronger expression. **B)** K-medoids clustering of 1354 genes showed in A) panel. In contrast to the log-transformed expression value used in the heat map, normalized read count values were used in this k-medoids clustering to allow visualization in better resolution. All genes were split into 24 clusters based on their trend of expression (which was already mean-centered) across the 4 positions of sex spectrum (WT female, Line A male, Line B male, WT male). These clusters were subsequently grouped into 5 categories based on their dimorphic properties (see main text for details). *Dsx1* was identified as a non-binary male-biased gene in this analysis (bottom right graph).

Total 12 RNA sequencing libraries were generated from triplicate of each of the four sexual phenotypes. We first focused on those that differentially expressed (DE) between wildtype male and wildtype female. With the cut-off threshold of p-value < 0.05 and absolute fold change ≥ 1.2 (Empirical analysis of Digital Gene Expression (EDGE) test), 1354 DE genes were identified and we found that *Dsx1* also belonged to this list (Fig 4A). To further categorize these genes based on their expression profile along the sex spectrum, we performed k-medoids clustering using normalized expression values (reads per million). As a result, we found 505 genes (37.3%) showing female-biased expression and 226 genes (16.7%) were male-biased. Interestingly among these genes, 449 (33.2%) showed non-binary expression profile, in which their activity seemed to be at intermediate level in intersex samples. These cases were referred to as non-binary genes, and the majority of them (398 genes) belonged to female-biased group. Other genes whose expression did not seem to be affected in intersex animals and remained comparable among the male groups regardless of *Dsx1* mutation types were referred to as binary genes (282 genes). *Dsx1* was detected as a male-biased and non-binary gene in this analysis, which is consistent with previous qPCR data. We disregarded the remaining 623 genes (46%) either because the difference between male and female were too small (even compared to that of *Dsx1*) or because their expression could not be correlated with the sex spectrum (Fig 4B).

**Fig 5:**
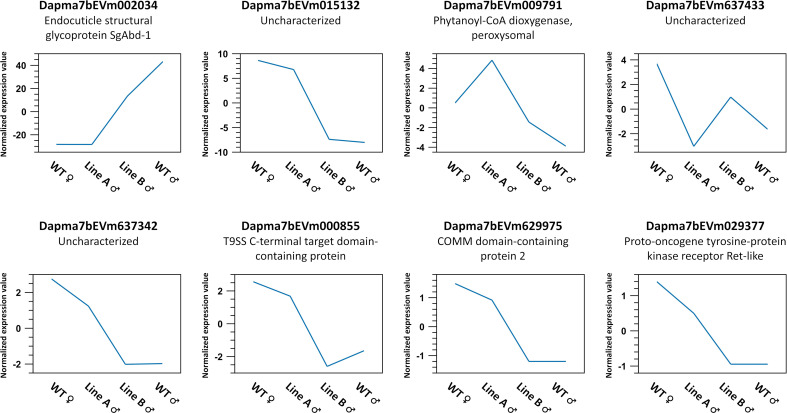
Eight genes showing differential expression between Line A male and Line B male. These genes were identified by applying a filter of p-value < 0.05, absolute fold change ≥ 1.2 (EDGE test) in Line A male vs. Line B male comparison to the list of 731 sex-biased genes. Graphs have same meaning as those from K-medoids clustering in Fig 4B.

Unexpectedly, from the list of 731 sex-biased genes above, we could find only 8 genes showing significant difference between Line A male and Line B male (p-value < 0.05, absolute fold change ≥ 1.2, EDGE test) (Fig 5), which is a small number despite the distinct phenotypes of the two mutants.

### Biological functions of the female- and male-biased genes

**Fig 6:**
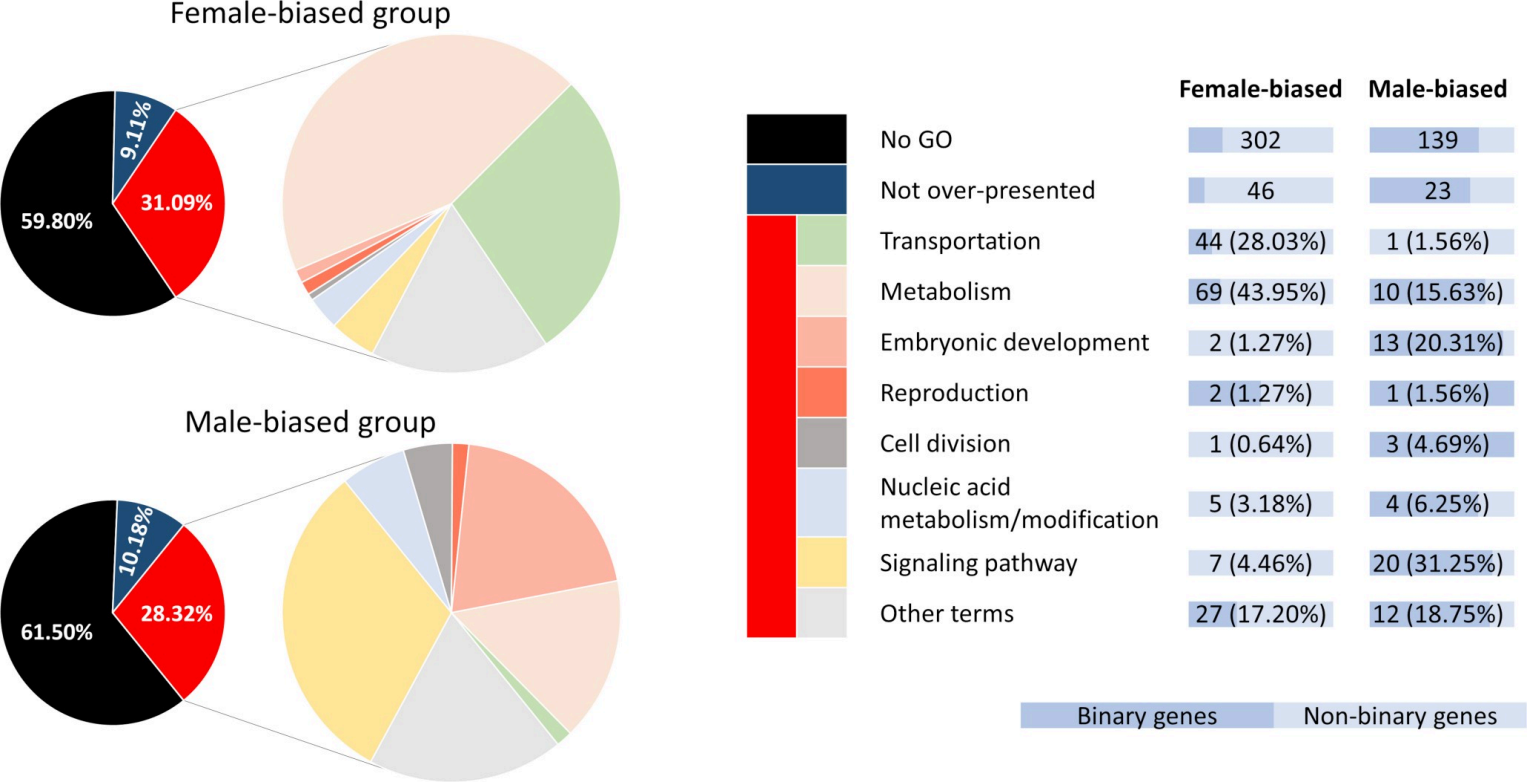
Number of genes assigned to each functional category described in S1 Fig. Aside from genes found to be accounted for over-presence of certain GO terms (red group), the majority from our gene list are not associated with any GO terms (black group) while some other genes did not cause significant enrichment of the terms they are annotated with (dark blue group).

A simple hypergeometric test were carried out to determine which Gene Ontology (GO) terms were enriched in the obtained gene collection. Our analysis showed that for the female-biased group, 109 GO biological process terms were over-presented while for the male-biased group this number was 176 (p-value < 0.05) (S1 Table). Using the parent-child relationship of GO terms, all over-presented terms could be classified into 8 large categories: “embryonic development,” “reproduction,” “cell division,” “metabolism,” “transportation,” “signaling pathway,” “nucleic acid metabolism/modification,” and “other terms.” In each category, clear difference between female-biased and male-biased group can be seen. The number of genes that were associated with “transportation” and “metabolism” in female-biased group were 113 (22.4%), much larger than that of the male-biased group which was 11 (4.9%). The opposite was found for “signaling pathway” and “embryonic development” categories (9 genes, 1.8% in female-biased against 33 genes, 14.6% in male-biased). We also noticed some common features between the two groups, for example chitin metabolism and several RNA-related gene expression regulation processes (S1 Fig). However, the majority (around 60%) of the genes in our list could not be annotated with any GO term. Distribution of binary and non-binary genes among functional categories was rather even, resulting in dominance of non-binary genes in all female-biased categories and of binary genes in all male-biased categories (Fig 6, S2 Table).

### Validation of RNA-seq gene expression with qPCR

**Fig 7:**
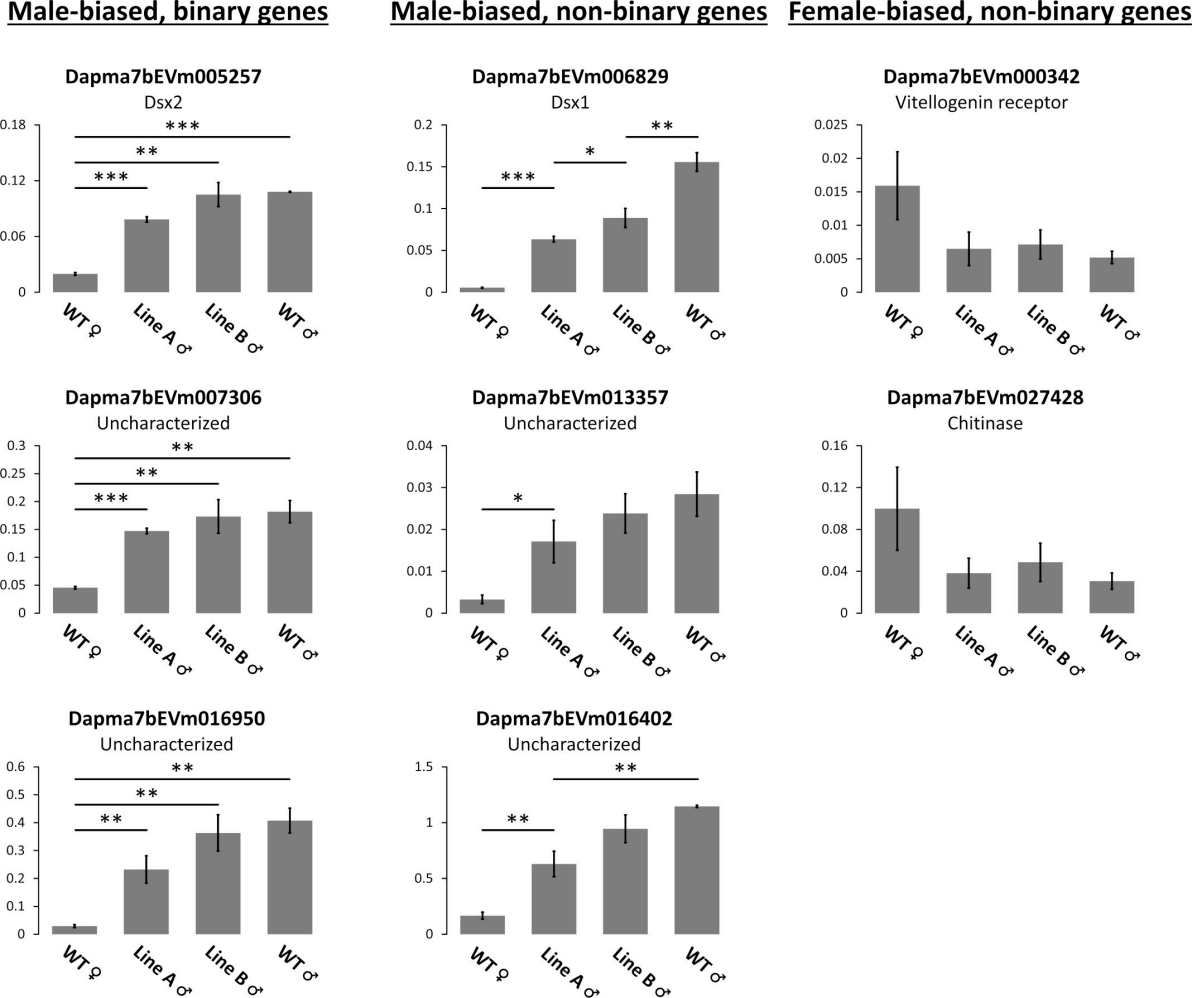
qRT-PCR of selected genes for validating RNA-seq expression. RNA samples of 40h embryos with triplicate for each group were used. All graphs show relative expression by normalization using the housekeeping ribosomal protein L32 gene. Error bars = SEM. * P < 0.05, ** P < 0.01, *** P < 0.001 (t-test).

To validate our RNA-seq data, we picked several genes for qRT-PCR analysis. The same RNA samples that we prepared for RNA-seq were used in this experiment. Three genes from “male-biased, binary” set, 3 genes from “male-biased, non-binary” set, and 2 genes from “female-biased, non-binary” set were chosen. Among them are *Dsx1*, the mutated gene in this study, and *Dsx2*, another DM-domain gene of which male-biased expression has already been well described in *D*. *magna* [28]. Dapma7bEVm007306, an uncharacterized protein, was chosen because this gene is located at the same locus and downstream of *Dsx1* and *Dsx2*. Three other genes, Dapma7bEVm013357, Dapma7bEVm016950 and Dapma7bEVm016402 (all are uncharacterized genes) are also found very close to each other in the same chromosome region (S2 Fig). Dapma7bEVm000342 (a vitellogenin receptor) and Dapma7bEVm027428 (a chitinase) were chosen because they are potentially the downstream elements of Dsx1 judging by their functions. Sex-bias could be confirmed in all cases while binary/non-binary pattern could be confirmed in 6/8 cases, indicating that our RNA-seq analysis is highly reliable (Fig 7).

## Discussion

Although sexual dimorphism can make male and female appear like two unrelated species, at genetic level, they differ only in genes on sex chromosome as in genetic sex determining species, or even in minuscule level if any as in environmental sex determining species. This similarity of genome between male and female may explain that sex change can happen naturally in several species [31,32]. These molecular and physiological evidence demonstrates that although sexual ambiguity is uncommon, it is a very natural manifestation of the genome and thus treating intersexes not as outliers but as an undeniable part of the population is very important. Our report here has confirmed that in one more species, *D*. *magna*, there are more to sex than just male and female and therefore the definition that sex is actually a spectrum can be applied to a much broader scale.

In contrast to extremely rare intersexuality in natural environment, we could develop a method to generate intersex phenotypes *D*. *magna* from the two modified genomes by simple chemical exposure. Previously, it was reported that intersex daphniids may appear under certain environmental setup such as high temperature [33]. However, non-genetic intersex-induction is inefficient and yields inconsistent result. Our approach, on the other hand, focused on disrupting the key sex determining factor Dsx1 of *D*. *magna* and therefore altered the entire sexual differentiation cascade downstream of Dsx1. Numerous studies have pointed out that DM-domain genes are involved in body wide somatic sexual development, from gonadal to non-gonadal and even neuronal tissues [19,34]. In *D*. *magna*, there are also evidence that Dsx1 is required for the establishment of testis and many other male-specific structures [28,29], which is consistent with our observation in this paper. Therefore, our modified *D*. *magna* strains represent intersex at whole body level and will serve as useful tools for the investigation into organogenesis of sexual traits as well as the creation of *D*. *magna* sex spectrum.

Our RNA-seq data revealed a set of 449 genes correlated with Dsx1 as their expression aligned to the sex spectrum given by our mutants (Fig 4B), suggesting that these genes are downstream factors of Dsx1. Interestingly, the majority of these genes are negatively correlated (398 genes), which means they are directly or indirectly repressed by Dsx1, hence suggest an important role of Dsx1 as a transcriptional repressor in addition to a transcriptional activator. Another set of 282 genes exhibited sex-biased activity but their dimorphism was unaffected in the mutants (Fig 4B). To explain, there are three possibilities. 1) These binary genes are less sensitive to Dsx1 dosage than non-binary genes. 2) Genes annotated with categories related to transcription factor activity in the male-biased group function upstream of Dsx1. Epistatic relationship between Dsx1 and these genes must be analyzed by their silencing, overexpression, and identification of Dsx1 binding sites. 3) These genes are under the control of another sex development pathway independent of Dsx1, as in case of *Drosophila* the *fruitless* branch is recruited to establish sexual behavior in parallel with *doublesex* branch [35]. However, this is rather unlikely in *D*. *magna* because *Dsx1* RNAi induced complete feminization including production of offspring [28,36].

In the RNA-Seq analysis, female-biased group seemed to invest more to “transportation” and “metabolism” with the enrichment of many amino acid and carbohydrate biosynthetic processes. Male-biased group on the other hand showed the upregulation of a variety of “signaling pathways”. This result suggested that Dsx1 as a transcription factor had activated multiple signaling pathways to redirect organogenesis to male development, while at the same time as a repressor had slowed down metabolism. Because the majority (around 60%) of the genes in our list could not be annotated with any GO term (Fig 6, S2 Table), investigation into these unknown genes is necessary before any conclusion about Dsx1 mode of action can be made.

Line A has a mutation of 6-bp deletion at start codon of *Dsx1* ORF and shows more severe feminization than Line B which harbors an intact *Dsx1* gene (Fig 2). Ribosomal scanning model suggests that deletion of the original start codon will force the translation to start from the second AUG, which may give rise to a mutated product in Line A. In this case, roughly 8% of amino acids will be truncated from N-terminus but the two conserved domains, DM-domain and oligomerization domain, will still remain intact (Fig 1C). This may possibly alter the interaction of Dsx1 to its binding target or to associated proteins, which reduces the performance of Dsx1 as a transcription factor. As many studies on *Drosophila* have pointed out, Dsx^F^ and Dsx^M^ may work in complexes such as dimers or heterodimers with other proteins to exert their sex- and tissue-specific functions [37–39]. As a complex, activity can be stabilized [40] and different complexes control different set of genes. Another possibility is that the truncation does not affect Dsx1 activity but translation from an alternative start site lacking simulating sequences has reduced the amount of translated protein. In mammalian, there is evidence that the *Dmrt1* gene is not dominant but dose sensitive [41] and therefore it can be expected that further reduction of Dsx1 amount would amplify feminization effect. However, the situation can be much more complicated as translation initiated at suboptimal context is not only inefficient but may also lead to leaky scanning where other downstream AUGs will also be recognized at start sites [42]. This means in Line A, there is likely a mixture of active and inactive Dsx1 variants that compete with each other. To clarify the correct mechanism, direct qualification and quantification of Dsx1 protein with Western blot for example would be necessary.

Despite the evident difference in sexual phenotype between Line A and Line B (Fig 2), the number of gene differentially expressed between the two lines was very small (Fig 5). While these genes expressed at rather low level, there was one gene named Dapma7bEVm002034 (Endocuticle structural glycoprotein SgAbd-1) which seem to be more active than the others. Annotation of this gene suggesting its role in carapace formation, explaining why there was visible difference in rostrum and first antennae morphology between Line A and Line B males right at first swimming juvenile stage. Perhaps at the chosen stage, the Dsx1-related sex differentiation cascade is yet to be fully displayed. As a matter of fact, expression of Dsx1 is temporally controlled and it is possible that Dsx1 can target more genes in later stages as there is a surge in Dsx1 activity during the transition from juvenile to adult [29]. In addition, to analyze more details of Dsx1-dependent transcriptional profile, enrichment of Dsx1 positive tissues or cells would be needed.

Last but not least, the critical role of Dsx1 as the central sex determining factor in *D*. *magna* is once again confirmed by this study. Consistent with previously published data in which embryonic knock-down of *Dsx1* by RNAi induced male to female reversal of gonadal and non-gonadal tissues [28], very similar effect could be found in our established mutants. However, it is noteworthy that *Dsx2*, another DM-domain gene neighboring *Dsx1* that also has strong male-biased activity [28], was as well detected as a male-biased and binary gene in our analysis (Fig 7). Although the sex determining function of *Dsx2* was not proven by the similar RNAi experiment [28], a detailed expression analysis pointed out that *Dsx2* became more active toward the end of vitellogenesis cycle and during spermatogenesis, strongly suggest a distinctive role of *Dsx2* in the sexual development of *D*. *magna* [43]. Here, we do not eliminate the possibility that *Dsx1* activity was actually extinguished in Line A and it was other factors such as *Dsx2* that built up male-like features in Line A male.

In summary, this study present a unique strain of *D*. *magna* with intersex phenotype resulted from mutating a core sex-determining element. By characterizing the transcriptome along the sex spectrum consisted of four different sexual states, we could also identified a genetic constituent that might underlie sexual development in *D*. *magna*. It can be learnt from this study that even though evolution has created binary switches in the sex determination machinery, the downstream elements that directly involved in building up sexual traits may not necessarily follow the binary rule but can fluctuate and therefore give rise to a spectrum of sexual phenotype. We expect Line A and Line B obtained here, together with more intersex animals that may be discovered later, would become powerful tool for the study of sex spectrum.

## Materials and methods

### *Daphnia* strains and culture conditions

Both Line A and Line B were generated using a transgenic strain of *Daphnia* expressing GFP ubiquitously throughout the body [44] as described previously [29]. To maintain the *Daphnia* population in asexual cycle, we used an optimal culturing condition in which 40 females were kept in 2.5 L ADaM [45], fed daily with 8×10^8^ *Chlorella vulgaris* cells and juveniles are removed every one or two days. Culturing room was set at 22–24°C and under 16:8 h light-dark cycle. For fluorescence photographing, we fed 0.3 mg dry yeast for one daphniid daily instead of algae to avoid their autofluorescence usually found in the gut.

### Production of male daphniids

Male production in *D*. *magna* can be induced by exposure to juvenile hormone analogs [30]. Here, mature females 18 h before oviposition were selected, moved to medium containing 1 μg/L fenoxycarb (Wako Pure Chemicals, Osaka, Japan) and cultured for 16 h. Embryos obtained after treatment would become males with 100% efficiency. Intersex ‘males’ of the mutants were also created using this method.

### Crossing of *Daphnia* strains

Crossing between strains requires sexual reproduction, which is inducible by putting the population under high density and starvation. In detail, we mixed 25 young males of Line B with 175 young females of Line A in one beaker containing 1 L ADaM and fed with 4.8×10^8^ *Chlorella* cells daily. Ephippia were harvested after 3 weeks of culturing and stored in dark at 4°C for one year.

To trigger the hatching, the ephippia were first air-dried overnight at room temperature, followed by rehydration in ADaM for 2 hours [46]. All ephippia were then opened to retrieve the resting eggs inside [47]. We incubated these activated resting eggs at standard culturing condition for one week and collected all hatched juveniles for genotyping. Genotyping method was the same as screening for mutants [29].

### Sampling of daphniids and RNA extraction

For samples that were not used in RNA-seq experiments, 20–30 embryos of the same clutch or 2–3 adults of the same age were collected as one sample with triplicate for each group. For samples used in RNA-seq, because a larger amount of RNA was needed, we pooled 40 h embryos from three mothers as one sample, also with triplicate for each group. As it was difficult to find mothers that lay eggs at the same time, immediately after oviposition, those releasing eggs earlier were kept in 4°C to wait for the others. This short-term ice-chilled preservation can efficiently suppressed biological processes such as hardening of egg shell and cytokinesis for up to two hours without damaging the *D*. *magna* eggs [48]. Once all ovulated mothers were obtained (typically in less than one hour for each group), the daphniids were returned to normal condition and cultured for 40 hour before subjected to dissection and isolation of embryos. Using this method, we could ensure that all embryos in one sample were perfectly synchronized at the time of sampling.

Immediately after collecting, all samples were transferred to liquid nitrogen for flash-freezing. RNA extraction was performed shortly after using Sepasol-RNAI solution (Nacalai Tesque, Kyoto, Japan), followed by phenol-chloroform purification and DNase treatment to remove genomic DNA contaminant.

### qRT-PCR

Total RNA was subjected to cDNA synthesis using random hexamers (Invitrogen) and SuperScript III Reverse Transcriptase (Invitrogen). For qPCR, PowerSYBR^®^ Green PCR Master Mix (Invitrogen) was used and reactions were carried out inside an Mx3005 P Real-Time PCR System (Agilent Technologies, CA, USA). The ribosomal protein L32 gene, whose whole body expression level was proven to be highly stable during development [49], was used for normalization of all other genes. Sequences of all qPCR primers can be found in S3 Table.

### RNA sequencing

We used the mRNA-seq service provided by Novogene (jp.novogene.com). According to the company, the submitted total RNA samples were first eluted through oligo(dT) columns to enrich mRNA. Then cDNA was synthesized using random hexamers followed by terminal repair and sequencing adaptor ligation to generate cDNA libraries. All 12 libraries passed their QC and were fed into Illumina sequencers for paired-end sequencing. Raw data filtering and adapter trimming were done as part of the service. What we received were clean data of ~10,000,000 read pairs per library and read length was 150 bp.

### RNA-seq data analysis

All analyses were done using the CLC Genomics Workbench software (Qiagen). Details about parameters that were used are as follows: 1) Mapping: mismatch cost = 2, insertion cost = 3, deletion cost = 3, length fraction = 0.8, similarity fraction = 0.8, global alignment = no, auto-detect paired distances = yes, strand specific = both, maximum number of hits for a read = 10, count paired reads as two = no, expression value = total counts, calculate expression for genes without transcripts = no, 2) Differential expression analysis (Empirical analysis of Digital Gene Expression (EDGE) test): total count filter cutoff = 5.0, estimate tagwise dispersions = yes, exact test comparisons = all pairs, 3) Heat map clustering: distance measure = Euclidean distance, cluster linkage = average linkage, filter settings = specify features, 4) K-medoids clustering: number of clusters = 24, distance metric = Euclidean distance, data level = group means, data to cluster = normalized expression values, subtract mean gene expression level = yes, 5) Annotation test (hypergeometric test): annotation to test = GO biological process, reduce feature set = no, values to analyze = normalized expression values, full set = *D*. *magna* 47,109 gene set, subset = female-biased or male-biased gene set. Assignment of GO terms to each gene was carried out using Blast2GO (BioBam). For mapping, we used the 2010 *D*. *magna* genome assembly v2.4 (GenBank accession LRGB00000000) together with the 2014 *D*. *magna* evg7f9b gene set as reference [50]. This gene set contains 29121 gene loci together with 17228 “culled” transcripts which may as well contain some valid loci. Among these genes, some are split-mapped, meaning that they consist of two fragments that mapped to distant positions in the genome assembly. Because these split-mapped genes disturb the visualization of gene distribution on the genome by the CLC Genomics Workbench software, we edited the gene set and treated the split fragments as two different genes. As a result, there are total 47,109 genes in the full set of our analysis. Also, although the original ID of each gene is in the format [Dapma7bEVm][6 digits], we added 7 characters indicating the scaffold/contig the gene is mapped to and 7 characters indicating its split attribute to make new unique IDs as can be seen in S2 Table.

### Statistical analysis

Processing of qPCR data was done using Microsoft Excel 2016. Mean values were obtained using the “average” function. SEM (standard error of the mean) values were obtained by dividing the standard deviation (obtained using the “stdev” function) by the square root of number of samples. For pair-wise statistical comparison, first an F-test was done by using the “f.test” function (only p-value will be calculated) to determine whether the variance of the two groups are equal or unequal. Since most pairs showed unequal variance (p-value threshold of 0.05), one-tail t-tests for two sample with equal variance were performed for all pairs by using the “t.test” function (only p-value will be calculated). Charts are generated also by using Excel in combination with PowerPoint.

## Supporting information

S1 Fig Grouping of the over-presented GO terms from S1 Table based on their hierarchical relationship. Panel **A)** are terms enriched in female-biased group and panel **B)** are terms enriched in male-biased group. Start of arrows indicates child end and end of arrows indicate parent end. All terms can be generally split into 8 large categories, “embryonic development”, “reproduction”, “cell division”, “metabolism”, “transportation”, “signaling pathway”, “nucleic acid metabolism/modification” and “other terms”, shown in different colors in this figure. This figure was manually constructed with reference to the QuickGO tool (www.ebi.ac.uk/QuickGO/).
(TIF)

S2 Fig Two gene clusters showing sex-specific expression. Dapma7bEVm006829 (*Dsx1*) and Dapma7bEVm005257 (*Dsx2*) are two DM-domain genes whose male-biased expression has been well described previously. These two gene can be found next to each other on scaffold02190 of *D*. *magna* genome. According to our data, Dapma7bEVm007306, another gene located downstream of *Dsx2*, also showed male specific expression. A similar case was found on scaffold00687 where three neighboring genes, Dapma7bEVm016402, Dapma7bEVm016950 and Dapma7bEVm013357, also shared male-biased expression (see main text and Fig 7 for qPCR result). Be noted that Dapma7bEVm007396 is originally split-mapped on contig10705 and scaffold01223. However when comparing with *D*. *pulex* genome, we judged that these two fragments could be mapped to a long poly-n region between *Dsx2* and Dapma7bEVm007899, resulting in the compiled version of scaffold02190 as shown in this figure.
(TIF)

S1 Table List of over-presented GO biological process terms for female-biased gene group and male-biased gene group. This list was obtained by performing hypergeometric tests using the sex-biased gene list as subset (505 genes in female set, 226 genes in male set) and *D*. *magna* 40,000+ gene collection as full set. Only terms with p-value < 0.05 are shown in this table.
(XLSX)

S2 Table The list of 731 sex-biased genes together with their raw and analyzed data, separated into four groups according to our clustering analysis. Each gene is also colored based on their annotation using the color scheme from Fig 6.
(XLSX)

S3 Table List of primers used in qRT-PCR. (XLSX)
