# Supplementary figures and images for: Two Doublesex1 mutants revealed a tunable gene network underlying intersexuality in Daphnia magna
Source: PLoS One. 2020 Aug 31;15(8):e0238256. doi: 10.1371/journal.pone.0238256 (PMC7458346; doi:10.1371/journal.pone.0238256)

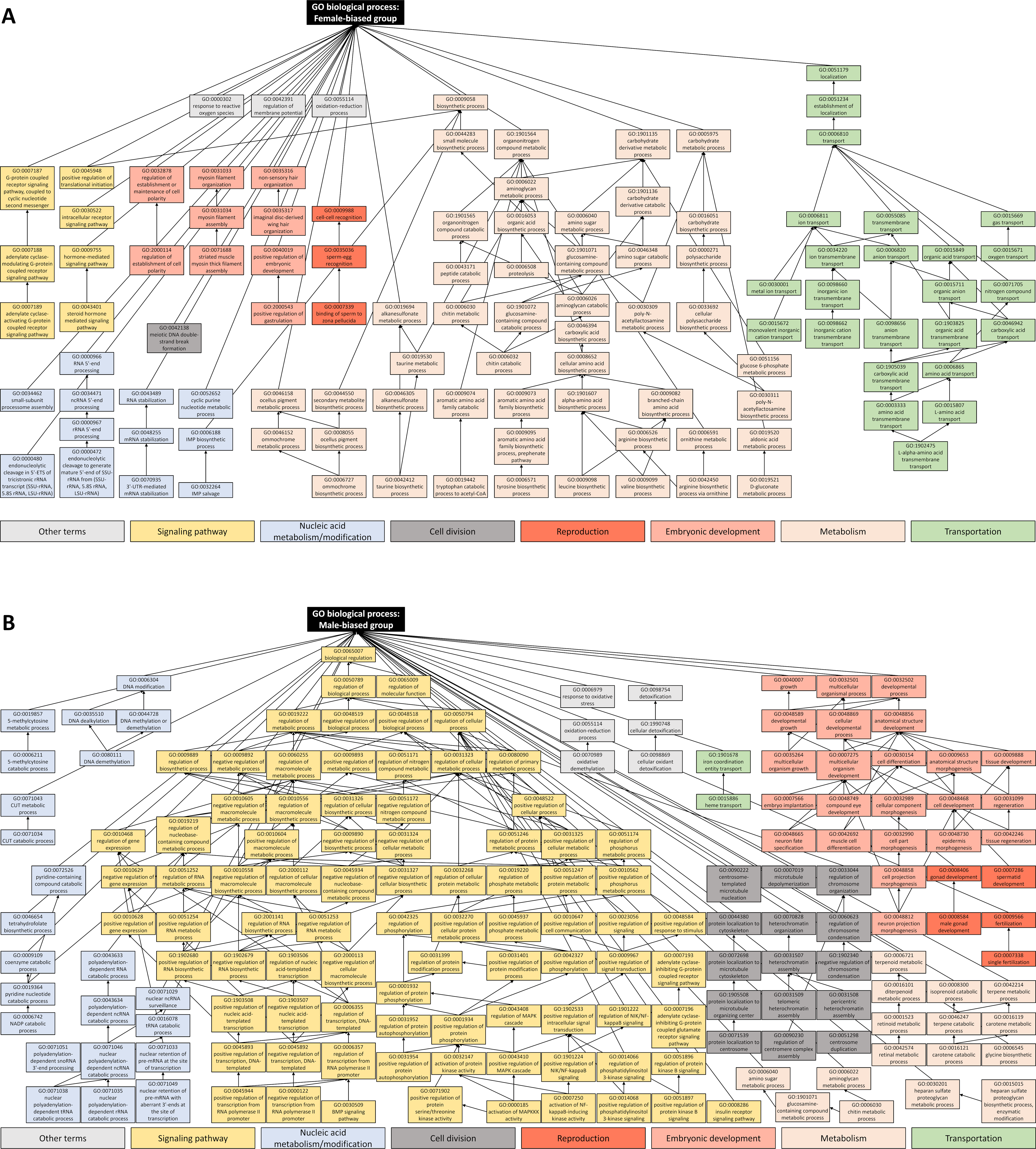

Supplement: S1 Fig — Panel A) are terms enriched in female-biased group and panel B) are terms enriched in male-biased group. Start of arrows indicates child end and end of arrows indicate parent end. All terms can be generally split into 8 large categories, “embryonic development”, “reproduction”, “cell division”, “metabolism”, “transportation”, “signaling pathway”, “nucleic acid metabolism/modification” and “other terms”, shown in different colors in this figure. This figure was manually constructed with reference to the QuickGO tool (www.ebi.ac.uk/QuickGO/). (TIF) [file pone.0238256.s001.tif]

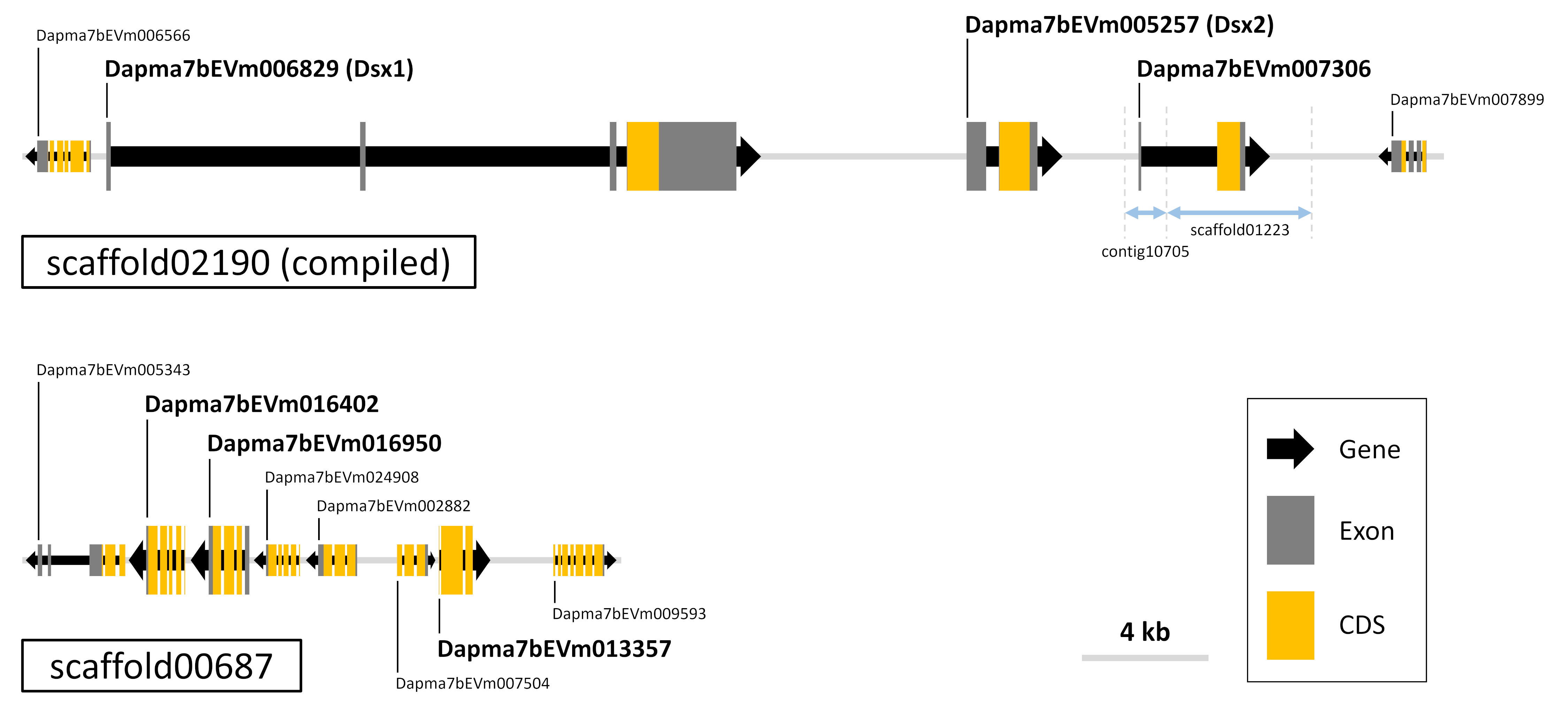

Supplement: S2 Fig — Dapma7bEVm006829 (Dsx1) and Dapma7bEVm005257 (Dsx2) are two DM-domain genes whose male-biased expression has been well described previously. These two gene can be found next to each other on scaffold02190 of D. magna genome. According to our data, Dapma7bEVm007306, another gene located downstream of Dsx2, also showed male specific expression. A similar case was found on scaffold00687 where three neighboring genes, Dapma7bEVm016402, Dapma7bEVm016950 and Dapma7bEVm013357, also shared male-biased expression (see main text and Fig 7 for qPCR result). Be noted that Dapma7bEVm007396 is originally split-mapped on contig10705 and scaffold01223. However when comparing with D. pulex genome, we judged that these two fragments could be mapped to a long poly-n region between Dsx2 and Dapma7bEVm007899, resulting in the compiled version of scaffold02190 as shown in this figure. (TIF) [file pone.0238256.s002.tif]
